# Improving Early Detection of Developmental Dysplasia of the Hip: A Study of Compliance With England’s Screening Programme

**DOI:** 10.7759/cureus.99864

**Published:** 2025-12-22

**Authors:** Atif Ghafar, Kishan Manani, Elhussein M Elgengehy, Mahmuda Rahman

**Affiliations:** 1 Orthopaedics and Trauma, Lancashire Teaching Hospitals, Preston, GBR; 2 General Practice, Royal Bolton Hospital, Bolton, GBR

**Keywords:** developmental dysplasia of the hip (ddh), hip screening compliance, infant hip screening, neonatal hip screening, newborn and infant physical examination, ultrasound scan referrals

## Abstract

Background

In England, the rate of late-diagnosed developmental dysplasia of the hip (DDH) is higher than that in neighbouring countries, suggesting potential gaps in the national screening pathway. This study aimed to evaluate adherence to the NHS Newborn and Infant Physical Examination (NIPE) programme guidelines to identify areas for improvement.

Methods

A retrospective, single-centre evaluation was conducted in a neonatal unit in England for all consecutive neonates born in August 2023. Main outcome measures included compliance with the 72-hour timeframe for the initial hip examination, accuracy of ultrasound (USS) referral decisions, timeliness of USS completion, and the distribution of clinician workload.

Results

All infants (302/302, 100%) received a hip examination within 72 h (median 21.0 h, IQR 13.3-28.0). Guideline-concordant referral decisions were 291/302 (96.4%; 95% CI 93.6-98.0). Under-referral occurred in 2/27 (7.4%) of indicated cases; over-referral was 9/275 (3.3%) among non-indicated infants and constituted 9/34 (26.5%) of all scans. Hip USS was completed in 34/34 (100%) referred infants; 20/34 (58.8%) scans were completed within 4-6 weeks, 5/34 (14.7%) were early (<4 weeks), and 9/34 (26.5%) were late (>6 weeks). Workload was concentrated: the top quartile of examiners (8/32) performed 200/302 (66.2%) examinations.

Conclusion

This evaluation found excellent compliance with the initial newborn hip examination, yet important weaknesses emerged later in the screening pathway. Inaccurate referrals and uneven examiner involvement were observed alongside delays, with a significant number of infants scanned outside the recommended 4-6-week window. These deviations may reduce the opportunity for timely, non-invasive treatment. Strengthening practice locally could include reviewing the referral-to-scan pathway and establishing a consistent group of higher-volume, credentialed examiners to support accuracy and imaging capacity. Further multi-centre work is needed to assess wider applicability and impact on clinical outcomes.

## Introduction

Developmental dysplasia of the hip (DDH) encompasses a spectrum of abnormalities affecting the hip joint, ranging from mild instability to complete dislocation [1]. It is one of the most common congenital musculoskeletal conditions, with an estimated prevalence of approximately 1-3% among newborns in the United Kingdom [2]. Early detection and timely interventions are critical for optimal outcomes [3]. Studies indicate that over 95% of infants diagnosed and treated early can achieve normal hip function, avoiding long-term complications such as chronic pain, early-onset osteoarthritis (OA), and impaired mobility later in life [4,5]. Conversely, delayed diagnosis often necessitates more invasive surgical interventions and increases the risk of poor long-term outcomes.

Neonatal screening programmes are designed to detect DDH in its early stages, ensuring prompt treatment to minimise adverse outcomes [6]. In the United Kingdom, the NHS Newborn and Infant Physical Examination (NIPE) programme provides the foundation for neonatal hip screening [7]. The protocol requires clinical assessments to be performed within 72 hours of birth and repeated at the 6-8-week postnatal check. These evaluations assess hip instability, restricted abduction, and risk factors such as breech presentation or a family history of DDH. Neonates with abnormal findings or risk factors are referred for a hip ultrasound (USS), which should be performed between 4-6 weeks of life. Despite these measures, England reports a late DDH diagnosis rate of 1.28 per 1,000 live births, substantially higher than that of neighbouring countries [8-12].

Numerous European countries achieve significantly lower rates of late DDH diagnoses through alternative screening strategies. Slovenia, for instance, implements universal hip USS screening alongside clinical examinations. Clinical assessments, including Ortolani, Barlow, and Galeazzi tests, are performed within the first few days of life. If findings indicate a stable hip with no risk factors, a follow-up USS and clinical examination are conducted at six weeks of age. However, for neonates with positive findings or risk factors, a USS is performed in the maternity ward, followed by orthopaedic consultation within two weeks if abnormalities are identified. This comprehensive approach has resulted in a late DDH diagnosis rate of just 0.09 to 0.23 per 1,000 live births [13].

Sweden, by contrast, uses a selective screening approach that combines clinical assessments with targeted USS referrals [14]. All newborns undergo an initial clinical examination for hip instability, including Ortolani and Barlow tests, performed by a paediatrician before discharge. Neonates with suspected instability or dislocation are referred to an orthopaedic surgeon, while all neonates, regardless of stability, receive follow-up assessments at child health centres at 6-8 weeks, six months, and 10-12 months of age. This structured approach has contributed to Sweden's low rate of late DDH diagnoses of 0.12 per 1,000 live births.

Despite the standardised adoption of the NIPE guidelines across the United Kingdom, significant regional variations in late DDH diagnosis rates exist. England reports a rate of 1.28 per 1,000 live births, while Scotland reports a slightly lower rate of 1.18 per 1,000 live births [10]. Notably, Northern Ireland achieves a much lower rate of 0.42 per 1,000 live births [11]. Variations in adherence to screening practices, rather than the guidelines themselves, may play a greater role in the differing late DDH diagnosis rates.

Against this background, this study examines key components of England’s neonatal and infant hip screening pathway in a single neonatal unit, focusing on process performance and potential opportunities for improvement in practice and capacity.

The objective of this single-centre service evaluation was to assess adherence to the NIPE hip screening pathway in a neonatal unit in England by describing completion and timing of the initial newborn hip examination; evaluating the accuracy of hip USS referral decisions against NIPE programme criteria; assessing the timeliness of USS completion in relation to the recommended 4-6-week window; and characterising the distribution of NIPE hip examination workload across clinician roles and individual examiners.

## Materials and methods

This retrospective observational study was conducted at a neonatal unit and included all babies born consecutively between 1 and 31 August 2023. Data collection occurred on 10 April 2025. All eligible records for the study period were identified from the unit’s NIPE register. Strict exclusion criteria were applied to maintain the integrity of the study population. Neonates born before 34 weeks' gestation, those admitted to the neonatal intensive care unit, those transferred to or from other hospitals, records with missing core data, and neonatal deaths were excluded. These criteria ensured the analysis focused on neonates receiving routine care under standard conditions.

Data were collected using multiple electronic systems to ensure comprehensive evaluation. The BadgerNet clinical system provided detailed neonatal records, including birth histories, documented risk factors, and clinician notes. The NIPE database was used to evaluate compliance with NIPE programme guidelines [7,15], specifically regarding the timing and documentation of hip screenings and hip USS referrals. Imaging data, including USS reports, were retrieved and verified through the picture archive and communication system (PACS) [16]. For each neonate, NIPE entries relating to hip screening and USS referral were cross-checked against BadgerNet documentation and, where applicable, the USS request and report within PACS to minimise misclassification of risk factors, indications, and outcomes. Reasons for delayed USS were not routinely documented and were therefore unavailable. This multimodal approach enabled a robust analysis of the screening process and the identification of areas requiring improvement.

When fields conflicted across, the study team manually reviewed the full clinical entry and the signed imaging report. A prespecified rule set privileged definitive and domain-specific sources (e.g., PACS for USS performance, timing and findings; NIPE for clinical hip examination findings and examiner role; BadgerNet for obstetric/neonatal risk factors and time of birth).

The primary process measure was completion of the initial hip examination within 72 hours of birth in line with the NIPE programme guidance. Reasons for any recorded delay (e.g., clinician availability, infant clinical status, documentation errors) were reviewed. USS referral decisions were assessed for guideline-concordance against NIPE criteria. A decision was classified as guideline-concordant if a USS was requested for any of the following indications: (i) breech presentation at ≥36 completed weeks; (ii) breech presentation at the time of birth between 28 weeks’ gestation and term; (iii) first-degree family history of hip problems in early life; or (iv) multiple pregnancies in which any NIPE hip risk factor was present or at least one baby had a positive hip examination. In addition, an abnormal clinical hip examination constituted an indication for USS irrespective of risk-factor status. An examination was classified as abnormal if any of the following were present: difference in leg length; knees at different levels when the hips and knees were bilaterally flexed; restricted unilateral limitation of hip abduction (a difference of 20 degrees or more between hips); gross bilateral limitation of hip abduction (loss of 30 degrees abduction or more); or a palpable ‘clunk’ on undertaking the Ortolani or Barlow manoeuvre. A normal hip examination was defined as the absence of these findings. Decisions outside the NIPE criteria were classified as not guideline-concordant and described as over-referral (USS without indication) or under-referral (no USS despite indication).

Variability among clinician groups performing these assessments was analysed to evaluate workload distribution and its potential implications for consistency and quality of care. Workload was described at both clinician and role levels (Foundation Year doctors, Advanced Neonatal Nurse Practitioners, midwives, and medical trainees).

Another key parameter was the timeliness of USS completion. According to guidelines, USS for neonates with positive examination findings or risk factors should be performed between four and six weeks of age. The study assessed adherence to this timeline and examined potential delays, including systemic inefficiencies and resource constraints.

Finally, the outcomes and management of neonates diagnosed with DDH were reviewed to ensure that all cases were appropriately managed and followed up.

Data analysis

Data from all neonates remaining after application of the prespecified exclusion criteria were entered into a password-protected Microsoft Excel (Microsoft Corporation, Redmond, USA) spreadsheet. The dataset included demographic and perinatal characteristics (sex, gestational age, birth weight), details of the hip examination (examiner role, timing, normal/abnormal findings), presence and type of NIPE-defined hip risk factors, USS referral decisions, USS timing and findings, and subsequent management and outcomes, including any late diagnoses of DDH.

Continuous variables (gestational age, birth weight, and times from birth to clinical examination and to USS) were summarised using medians and interquartile ranges (IQRs) and, where informative, means and standard deviations (SDs), as reported in the Results section. Categorical variables (for example, presence of recognised risk factors, referral status, guideline concordance of decisions, scan-timeliness categories, and clinician role) were summarised as counts and percentages with denominators stated.

Key process measures, including completion of the initial hip examination within 72 hours, accuracy of USS referral decisions, and completion of USS within the recommended 4-6-week window, were summarised as proportions with two-sided 95% confidence intervals calculated using the Wilson score method. USS referral decision accuracy was evaluated at the level of individual decisions and classified as guideline-concordant, over-referral, or under-referral. Workload distribution across clinician roles was described and formally assessed using a chi-squared goodness-of-fit test, comparing observed examination counts per role with those expected under an equal caseload assumption. Data were managed using Microsoft Excel for Microsoft 365 (Version 2408), and key statistical analyses, including calculation of Wilson score confidence intervals and chi-squared tests, were performed in R (version 4.4.2; R Foundation for Statistical Computing, Vienna, Austria). Statistical significance was defined a priori as p<0.05. All analyses were conducted on the final cohort after application of the prespecified exclusion criteria.

Ethical considerations were carefully addressed to maintain the integrity of the study. All data were anonymised, and access was restricted to authorised personnel to ensure compliance with confidentiality requirements. Formal research ethics committee approval was not required, as the study involved a retrospective analysis of data collected as part of a quality assurance process. This approach aligns with the guidance provided by the Health Research Authority, available at https://www.hra-decisiontools.org.uk/research/result7.html.

## Results

Study population

There were 362 neonates identified during the study period (August 1, 2023-August 31, 2023) in a single neonatal unit in England. In accordance with the prespecified exclusion criteria, 60 records were excluded owing to incomplete documentation and inter-hospital transfer, leaving 302 neonates for analysis. Baseline characteristics are summarised in Table 1.

**Table 1:** Baseline characteristics of the analysed cohort (n=302) Percentages from the total cohort (n=302). IQR: Interquartile range

Characteristic	Value
Sex: male	173 (57.3%)
Sex: female	129 (42.7%)
Gestational age, median (IQR)	39+4 weeks (38+5–40+3)
<37 weeks	11 (3.6%)
37–38+6 weeks	78 (25.8%)
39–40+6 weeks	175 (57.9%)
≥41 weeks	38 (12.6%)
Birthweight, median (IQR)	3,408 g (3,104–3,770)
<2500 g	12 (4.0%)
2500–3999 g	251 (83.1%)
≥4000 g	39 (12.9%)

Initial hip assessment (primary outcome)

A newborn hip examination was documented for 302/302 (100%) infants. Time from birth to examination was available for all infants. The median time to examination was 21.0 hours (IQR 13.3-28.0 hours). Examination timing was: 66/302 (21.9%) at ≤12 hours; 123/302 (40.7%) at >12-24 hours; 98/302 (32.5%) at >24-48 hours; and 15/302 (5.0%) at >48-72 hours. No examinations occurred beyond 72 hours. The minimum recorded time was 5.0 hours, and the maximum was 70.0 hours (mean 22.6, SD 12.2).

Abnormal clinical findings at the initial examination were recorded in 2/302 (0.7%). Both were a palpable hip clunk. No other abnormal signs (for example, limited abduction or asymmetric skin creases) were documented.

Recognised screening risk factors were present in 27/302 (8.9%). Within this indicated subgroup, mutually exclusive categories were breech only (including breech at ≥36 weeks or breech twin) 18/27 (66.7%); first-degree family history only 6/27 (22.2%); breech + first-degree family history 1/27 (3.7%); and first-degree family history with a palpable hip clunk 2/27 (7.4%). Individual-level characteristics for these neonates are shown in Table 2.

**Table 2:** Characteristics of neonates with recognised risk factors or abnormal clinical hip examination findings (n = 27) Risk factors and abnormal hip examinations classified according to the NIPE programme criteria. NIPE: NHS Newborn and Infant Physical Examination

Neonate	Sex	Gestational age (weeks+days)	Birthweight (g)	Risk factor(s)/Abnormal exam
1	Male	38+0	2980	Breech at ≥36 weeks
2	Male	39+3	2860	Family history
3	Female	39+2	3675	Breech at ≥36 weeks
4	Female	39+1	4160	Breech at ≥36 weeks
5	Female	39+4	4250	Family history
6	Female	38+4	3100	Breech at ≥36 weeks
7	Male	39+1	3230	Family history + Palpable hip clunk
8	Female	39+0	3370	Breech at ≥36 weeks
9	Female	35+4	1795	Breech at birth
10	Female	38+1	3220	Breech at ≥36 weeks
11	Male	39+0	3620	Breech at ≥36 weeks
12	Female	35+1	2130	Breech twin at birth
13	Male	39+4	4050	Breech at ≥36 weeks
14	Male	38+1	2900	Breech at ≥36 weeks
15	Male	39+0	3765	Family history
16	Male	41+2	3760	Breech at ≥36 weeks
17	Male	38+3	3600	Family history + Palpable hip clunk
18	Male	39+2	3570	Breech at ≥36 weeks
19	Male	42+0	3930	Breech at ≥36 weeks
20	Male	39+0	3960	Breech at ≥36 weeks
21	Female	35+0	2270	Family history
22	Male	40+0	3665	Breech at ≥36 weeks
23	Male	39+1	2620	Breech at ≥36 weeks
24	Female	37+1	2675	Breech at ≥36 weeks
25	Male	40+2	3550	Family history
26	Male	40+3	2910	Family history + Breech at ≥36 weeks
27	Male	40+3	3085	Family history

USS referral decisions and decision accuracy

Referral status was recorded for all infants (302/302, 100%). Among those with an indication for USS (n=27), 25/27 (92.6%) were referred. Across the cohort, 34/302 (11.3%) infants were referred for USS, 25/34 (73.5%) with a documented guideline indication and 9/34 (26.5%) without an indication. Among infants without an indication (n=275), 9/275 (3.3%) were nevertheless referred (over-referral).

USS decision accuracy was assessed at the decision level across the cohort. Overall, 291/302 (96.4%; 95% CI 93.6-98.0) decisions were according to guidelines. Within the indicated subgroup, 25/27 (92.6%; 95% CI 76.6-97.9) decisions met guideline standards and 2/27 (7.4%; 95% CI 2.1-23.4) represented under-referral. Referral outcomes for indicated neonates are shown in Table 3. Within the non-indicated subgroup, 266/275 (96.7%; 95% CI 93.7-98.3) decisions met guideline standards and 9/275 (3.3%; 95% CI 1.7-6.1) represented over-referral. Among the nine over-referrals, reasons were no documented indication 5/9 (55.6%); documentation errors (false risk factors: breech at delivery or family history of DDH) 2/9 (22.2%); and an unrelated condition (clubfoot) 2/9 (22.2%).

**Table 3:** Referral outcomes for indicated neonates (n = 27, mutually exclusive categories) Percentages in the “Referred” and “Not referred” columns are calculated within each indication category (row). NIPE: NHS Newborn and Infant Physical Examination

Indication (NIPE programme-defined)	Total in subgroup, n (%) of indicated	Referred, n (%)	Not referred, n (%)
Breech only (≥36 weeks or breech twin)	18 (66.7%)	17 (94.4%)	1 (5.6%)
Family history only	6 (22.2%)	5 (83.3%)	1 (16.7%)
Breech + Family history	1 (3.7%)	1 (100%)	0 (0%)
Family history + Palpable hip clunk	2 (7.4%)	2 (100%)	0 (0%)
Total	27 (100%)	25 (92.6%)	2 (7.4%)

Scan completion and timeliness of USS

USS completion was documented for all referred infants. A scan was performed in 34/34 (100%) referred cases, comprising 25/25 (100%) within the guideline-indicated subgroup and 9/9 (100%) within the over-referral subgroup. Time to scan was available for all completed scans (34/34, 100%).

**Figure 1:**
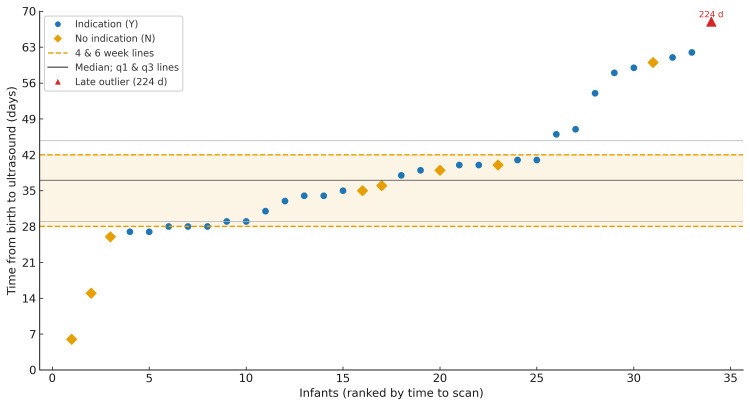
Time from birth to hip USS among referred infants (n=34) The 4–6-week window is highlighted; a 224-day outlier is shown as an outlined, labelled inset. X-axis:  ≤70 days USS: Ultrasound

Using the NIPE programme standard of completion within 4-6 weeks from birth, 20/34 (58.8%; 95% CI 42.2-73.6) scans met the standard; 5/34 (14.7%) occurred before four weeks (non-compliant early) and 9/34 (26.5%) occurred after six weeks (non-compliant late). The median time to USS was 37 days (IQR 29-45; range 6-224). The distribution of time from birth to USS for referred infants is shown in Figure 1.

Workload distribution

Clinician identifier (role plus letter; anonymised) was recorded for all assessments (302/302, 100%). Across the study period, 32 individual clinicians completed at least one newborn hip examination. The per-clinician caseload had a median of four assessments (IQR 2-13; range 1-53). The five highest-volume clinicians accounted for 153/302 (50.7%) assessments; the top eight for 200/302 (66.2%); and the top 10 for 225/302 (74.5%).

**Figure 2:**
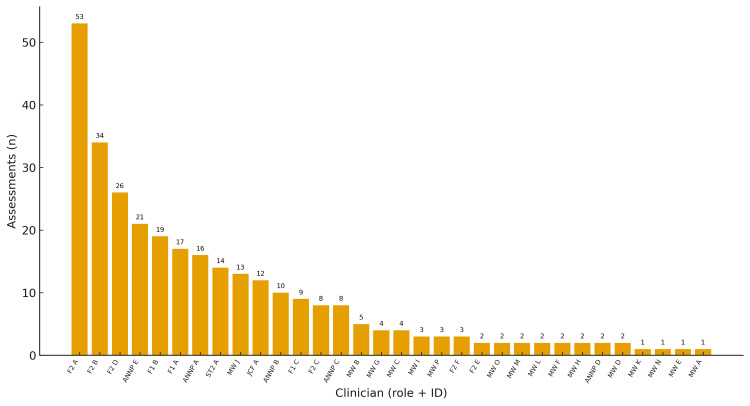
Per-clinician caseload of newborn hip examinations (n=302 assessments across 32 clinicians) Bars show the total number of examinations per anonymised clinician, labelled by role and identifier (e.g., F2 A). F1/F2: Foundation Year One/Two doctors; ANNP: Advanced Neonatal Nurse Practitioner; MW: Midwife; ST2: Specialty Trainee Year 2; JCF: Junior Clinical Fellow. Letter suffixes (A, B, etc.) denote anonymised clinician.

At the role-group level, assessments were distributed as follows: F2 126/302 (41.7%); ANNP 57/302 (18.9%); MW 48/302 (15.9%); F1 45/302 (14.9%); ST2 14/302 (4.6%); and JCF 12/302 (4.0%) (F1/F2, Foundation Year One/Two doctors; ANNP, Advanced Neonatal Nurse Practitioner; MW, Midwife; ST2, Specialty Trainee Year 2; JCF, Junior Clinical Fellow). Workload distribution differed from expectation: χ²(5)=170.0, p<0.0001. Within roles, the number of distinct clinicians contributing was: F2 (6), F1 (3), ANNP (5), MW (16), ST2 (1), and JCF (1). Per-clinician caseloads are displayed in Figure 2.

Outcomes

USS findings and clinical outcomes are summarised in Table 4. USS was performed for all referred infants, and no infant had sonographic evidence of DDH at the initial USS. Across the whole cohort, no late diagnoses of DDH (defined as diagnosis >12 weeks from birth) were recorded during the study period.

**Table 4:** Ultrasound findings and clinical outcomes Late DDH diagnosis: diagnosis of DDH >12 weeks from birth. DDH: Developmental dysplasia of the hip; USS: ultrasound

Outcome	Population	n/N	%
USS performed	Referred infants	34/34	100
DDH on initial USS	Referred infants	0/34	0
Late DDH diagnosis	Whole cohort (all infants)	0/302	0

## Discussion

This evaluation of England’s NIPE hip screening programme reveals a critical paradox: while initial newborn examinations demonstrated excellent compliance, significant downstream failures in referral accuracy and diagnostic timeliness threaten to undermine the programme’s overall effectiveness. Our findings highlight vulnerabilities in the screening pathway that align with broader concerns raised in national audits [8,9].

A key strength identified was the 100% compliance with performing the newborn hip examination, underscoring the unit’s commitment to this core standard. All examinations were completed well within the 72-hour national guideline. A majority (62.6%) were performed within 24 hours of birth, while the remaining 37.4% occurred between 24 and 72 hours. While fully compliant, this variability is a common feature in busy units, often reflecting operational pressures rather than clinical need [17].

The overall referral accuracy of 96.4% appears high, yet the specific errors reveal interconnected system pressures. The under-referral rate of 7.4% in the indicated group, while small in number, represents a significant process failure, as these are the infants the programme is specifically designed to catch [18]. Conversely, while the over-referral of infants without an indication was only 3.3%, these cases accounted for 26.5% of all infants referred for an USS. In effect, more than a quarter of the USS service’s workload for this cohort was clinically unnecessary, placing a direct and avoidable strain on diagnostic capacity. Each inappropriate referral consumes a valuable appointment slot that could otherwise be used for an indicated infant, which could plausibly displace imaging capacity and cause avoidable parental anxiety [19].

A pivotal finding of this study is the poor compliance with the NIPE programme’s recommended 4-6-week window for diagnostic USS, a failure point that resonates with national reports [7,8]. Our observation that only 58.8% of infants were scanned within this target period is a critical concern. The 4-6-week target is evidence-based; scanning before four weeks can produce high false-positive rates due to physiological immaturity that often resolves spontaneously [20]. Delays beyond 6-8 weeks are more perilous, as the efficacy of the Pavlik harness declines, increasing the likelihood of requiring more invasive surgery [6,21]. The extreme delays, including one outlier at 224 days, highlight that, even with excellent initial examination rates, downstream bottlenecks in diagnostic services can fundamentally undermine a screening programme’s effectiveness, a challenge that is likely systemic rather than unit-specific [8]. In this cohort, no DDH cases were identified; accordingly, our data cannot demonstrate the downstream effects of scan timing on diagnosis or treatment. Moreover, the absence of routinely recorded reasons for delay precludes attribution of specific causes for late or early scans.

The highly skewed workload distribution identified in this study suggests that a dedicated core-team model for NIPE hip assessments may be worth exploring. Our results show a stark disparity, with the top 25% of clinicians performing two-thirds of all examinations, while a significant proportion performed only one or two. This pattern risks skill degradation among low-volume practitioners, as proficiency in clinical skills is closely tied to volume and repetition [22,23]. UK professional standards also mandate that clinicians maintain competence through appropriate experience, supervision, and audit [24]. A more robust and potentially safer model may be to restrict the examination to a core team of high-volume, accredited practitioners. This approach has been shown to support more consistent skill application, simplify quality assurance, standardise referral thresholds and strengthen the screening pathway [8,18].

Limitations

The findings should be interpreted in the context of several limitations. As a single-centre evaluation, the results may not be generalisable; therefore, larger, multi-centre studies are warranted to determine if these specific process failures are widespread across the NHS. The retrospective nature of the study means that the analysis was dependent on the completeness and accuracy of existing clinical documentation, and misclassification of risk factors or examination findings cannot be excluded. In addition, each neonate was examined by a single clinician, and no duplicate or blinded second examinations were performed; consequently, inter-rater reliability for hip examinations could not be assessed, and our findings reflect documented interpretations of the individual clinician performing the examination. Sixty records were excluded for prematurity (<34 weeks), inter-hospital transfer, or incomplete core documentation. These exclusions were intentional, to focus inference on neonates receiving routine care under standard NIPE conditions; however, they may introduce selection bias. Reasons for delayed USS were not routinely recorded, limiting attribution of specific causes of delay. No cases of DDH were identified in our cohort. While this is a positive outcome, it means we cannot directly correlate the identified process failures with adverse clinical outcomes. The study's strength, however, lies in its detailed evaluation of the screening process and the identified gaps, which present tangible risks to patient safety that warrant urgent attention.

Implications for practice and policy

Based on the findings and within the context of these limitations, the implications are evidence-based and actionable. First, the shortfall in meeting the optimal 4-6-week USS window warrants urgent investigation. A collaborative root-cause analysis between neonatal and radiology teams should map current processes, identify bottlenecks, and streamline the referral-to-scan pathway. Timely diagnosis reduces the likelihood of complex surgical intervention, making pathway optimisation a patient-safety priority [18,20,21]. Second, the marked workload disparity may have implications for service delivery. Services may benefit from piloting a dedicated core team of higher-volume, credentialed examiners, consistent with principles of skill maintenance [22]. Concentrating activity among higher-volume practitioners is associated with more consistent performance and better outcomes in procedural care and could strengthen quality assurance and help standardise referral thresholds [23]. Multi-centre evaluation would help determine generalisability and impact on outcomes.

## Conclusions

This single-centre evaluation demonstrates excellent adherence to the initial newborn hip examination but highlights vulnerabilities later in the screening pathway. Small yet important referral errors and a skewed distribution of examiner workload coincide with shortfalls in delivering USS within the recommended 4-6-week window. Delayed or very early scans may reduce opportunities for timely, non-invasive treatment. Locally, a referral-to-scan pathway review and exploration of a dedicated core team of higher-volume, credentialed examiners may help reduce unnecessary referrals, protect USS capacity, and improve timeliness. Multi-centre work is warranted to determine generalisability and to assess the impact on clinical outcomes.
